# Incidental Hepatocellular Carcinoma after Liver Transplantation: Clinicopathologic Features and Prognosis

**DOI:** 10.3390/medicina59010030

**Published:** 2022-12-23

**Authors:** Fatih Ozdemir, Volkan Ince, Sertac Usta, Brian I. Carr, Harika G. Bag, Ayse Nur Akatli, Aysegul Sagir Kahraman, Sezai Yilmaz

**Affiliations:** Department of Surgery, Liver Transplantation Institute, Inonu University, Malatya 44210, Turkey

**Keywords:** incidental, hepatocellular carcinoma, liver transplantation

## Abstract

***Background*:** The prognostic impact and clinicopathologic features of incidental hepatocellular carcinoma (iHCC) detected in explanted livers of patients undergoing liver transplantation (LT) has been a controversial issue in previous studies when compared with patients who are diagnosed with hepatocellular carcinoma (pdHCC) before LT. We aimed to review and compare these patient groups in a high-volume LT center. ***Methods*:** The present study involves a retrospective analysis of 406 HCC patients who received LT between January 2002 and April 2022. Among these patients, demographic data, histopathologic features and prognosis for iHCC and pdHCC were evaluated. ***Results*:** In our series, 406 patients’ final diagnosis was HCC after they had received LT, nevertheless 54 patients in this HCC group were diagnosed incidentally after the pathological evaluation of the explanted livers. The etiology of the underlying liver disease between pdHCC (*n* = 352) and iHCC (*n* = 54) groups had some differences in our study population. Most of the patients in the pdHCC group had moderately differentiated tumors (45.7%). On the other hand, most of the patients in the iHCC group had well differentiated tumors (79.6%). There were 158 (44%) patients who met the Milan criteria in the pdHCC group while there were 48 (92%) patients in the iHCC group (*p* < 0.001). IHCC patients had statistically better 1, 3, 5 and 10 years disease-free and overall survival rates when compared with pdHCC patients. There was only 1 (1.8%) patient who had tumor recurrence in the iHCC group while 76 (21%) patients had tumor recurrence in the pdHCC group (*p* = 0.001). There is no disease free and overall survival difference when iHCC patients are compared with pdHCC patients who met the Milan criteria. ***Conclusion*:** It is the first study to show that iHCC patients may differ from pdHCC patients in terms of etiological features. IHCC tumors show better histopathologic features than pdHCC with low recurrence rate and iHCC patients have better survival rates than pdHCC patients.

## 1. Introduction

Hepatocellular carcinoma (HCC) is one of the major causes of death in patients with end-stage liver disease [1]. Liver transplantation (LT) can be the best therapy for patients who are within the Milan criteria, which is a single nodule less than 5 cm, or up to 3 nodules less than 3 cm each, without macrovascular invasion and extrahepatic spreading [2]. Tumor recurrence rates may be up to 20% (or more frequently) for patients beyond the Milan criteria [3]. Adequate patient selection, pre-transplantation locoregional therapies and prioritization within the waiting list can be done in order to prevent HCC recurrences [4]. “Incidental hepatocellular carcinoma” (iHCC) is a clinical condition, which is unexpectedly diagnosed after the histopathological evaluation of explanted livers in patients who have undergone LT for a reason other than tumors. Despite achievements due to radiologic evaluation, the diagnosis of iHCC in the explanted livers can still be a common condition for the patients undergoing LT for end-stage liver disease. Various prevalence rates were reported for iHCC (4.2% to 40%) [5,6,7,8,9]. Different prognostic factors of iHCC were reported in the previous studies [7,10,11]. In some studies, there is no prognostic effect either in disease-free survival (DFS) or in overall survival (OS); on the other hand, a worse prognostic effect can be observed in iHCC patients than “preoperatively diagnosed HCC” (pdHCC) groups [5,6,7,8,9,10,11]. We aimed to compare the clinicopathologic and prognostic features of pdHCC and iHCC patients who received LT at the Inonu University Liver Transplantation Institute. We hypothesized that iHCC patients have better clinicopathologic features and better prognosis.

## 2. Materials and Methods

The liver transplantation Institute of the Inonu University database, which is recorded prospectively for every LT consecutively, was queried retrospectively. This study was approved by Institutional Ethics Committee of Inonu University (Approval number: 2022/3931). The present clinical investigation has been managed according to the principles contained in the Declaration of Helsinki. Liver transplantations between January 2002 and April 2022 were reviewed (*n* = 3204). Patients with post-transplant follow up period less than 90 days and who transplanted for non-tumoral reasons were excluded, leaving 406 patients who have pathologically confirmed HCC to be included in the study. Of these, 54 were iHCC. Demographic features, underlying liver disease, Child–Pugh, MELD scores, pretransplant tumor marker alpha-feto protein (AFP), gamma glutamyl transferase (GGT) levels and explanted liver’s histopathological data of these patients were recorded. Number of nodules and the size of the tumors were described. Additionally, the grade and microvascular invasion of the tumors were recorded.

Liver transplantation is not recommended for patients with a MELD score of less than 15 unless there are clinical signs of liver decompensation. Cirrhotic patients were screened by liver ultrasound every 6 months according to international guidelines. If there was a suspected liver nodule detected by ultrasound or a raised AFP level, computed tomography (CT) and/or magnetic resonance imaging (MRI) was ordered pre-operatively. During post-transplant follow-up, serum AFP levels and imaging modalities, such as CT or MRI, were evaluated in order to diagnose recurrence at every four months for the first three years, and then the patients were checked for a recurrence every year by PET-CT scan. Recurrence rates, OS and DFS were assessed both for pdHCC and iHCC. Then, we compared the survival rates of patients within Milan in the pdHCC group versus iHCCs; lastly, we analyzed the survival rates of iHCC patients according to Milan criteria.

### Statistical Methods

Normal distribution of quantitative variables was assessed by Shapiro–Wilk test and summarized by median, minimum and maximum values. Comparisons of the two independent groups according to quantitative variables were performed by Mann–Whitney U test. Qualitative variables were expressed as count and percentage. For comparisons, Pearson’s chi-square, continuity-corrected chi-square tests and exact significance of Pearson’s test were used where appropriate. Additionally, for comparisons according to qualitative variables with more than two categories, Bonferroni adjustment was used for comparison of the column proportions of the two independent groups. Kaplan–Meier method and log-rank test were used for survival analysis. In all analyses, significance level was considered as 0.05.

## 3. Results

### 3.1. Demographic Features

The mean follow-up period of the 406 patients was 9.45 ± 0.38 (8.7–10.2 years, 95% CI). The average age of the pdHCC patients was 53.3 (4-72) and 49.4 (2-72) for iHCC patients. The male-to-female ratio was 303/49 for pdHCC and 46/8 for iHCC, respectively. The mean body mass index (BMI) of the patients for the pdHCC group was 26.2 (16.3–46.9) and also the mean BMI of iHCC group was 26.5 (16.4-41). The etiology of the underlying liver disease for pdHCC patients was 292 viral hepatitis (83%), 40 cryptogenic cirrhosis (11.4%), 5 Budd–Chiari syndrome (1.4%), 4 alcoholic liver disease (1.1%), 4 metabolic liver disease (1.1%), 2 NASH (0.6%) and other reasons for 5 patients (1,4%). On the other hand, the etiology of the underlying liver disease for iHCC patients was 32 viral hepatitis (59.3%), 12 cryptogenic cirrhosis (22.2%), 4 Budd–Chiari syndrome (7.4%), 2 alcoholic liver disease (3.7%), 2 metabolic liver disease (3.7%), 1 NASH (1.9%) and 1 other reason (hepatopulmonary syndrome) (1.9%). The average MELD score for pdHCC was 13.2 (6-41) and it was 17 (9-31) for iHCC. The CHILD scores of the patients were higher in the iHCC group. The pre-transplant median AFP values in the pdHCC group was 12 ng/mL (0.3-20179), and it was 4.45 ng/mL (0.4-1001) in the iHCC group. The demographic features were summarized at Table 1.

### 3.2. Histopathological Features of Incidental HCC and Preop Diagnosed HCC

There were 186 patients (52%) who had multinodular tumors in the pdHCC group, while there was an equivalent 18 (34%) patients in the iHCC group (*p* = 0.012). Median tumor diameter was 2 cm (0.1-24) in the pdHCC patients and it was 1 cm (0.1-2.5) in iHCC patients (*p* < 0.001). Most of the patients in the pdHCC group had moderately differentiated tumors (45.7%, *n* = 161). On the other hand, most of the patients in the iHCC group had well differentiated tumors (79.6%, *n* = 4 3) (*p* < 0.001). There were 169 (48%) patients who had no vascular invasion, while 135 (38.4 %) patients had microvascular invasion and 48 (13.6%) patients had macrovascular invasion in the pdHCC group. There were only four (7.4%) patients who had microvascular invasion in the iHCC group. There were 158 (44%) patients who met the Milan criteria in the pdHCC group, while there were 48 (92%) patients in the iHCC group who met it (*p* < 0.001) (Table 2).

### 3.3. Recurrence, Overall Survival and Disease Free Survival

**Figure 1:**
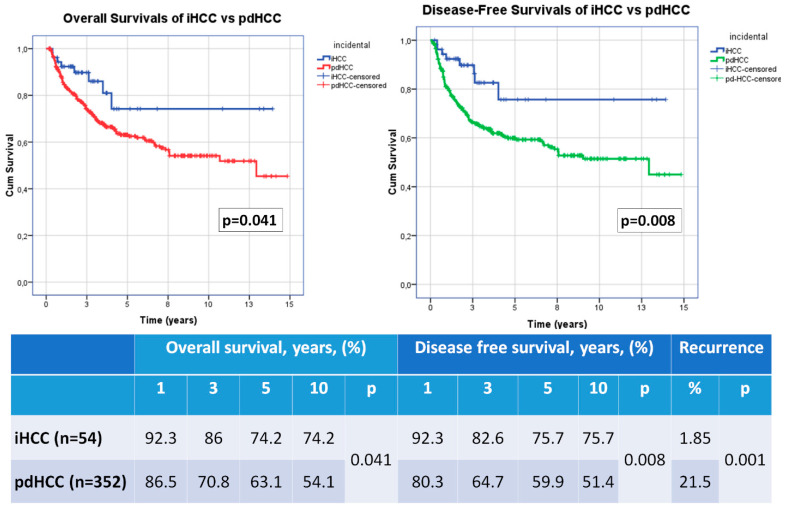
Survival comparison of Hepatocellular carcinoma patients (iHCC vs. pdHCC).

Firstly, we compared the survival and recurrence rates of the iHCC patients (*n* = 54) versus pdHCC patients (*n* = 352). The iHCC patients had statistically better 1, 3, 5 and 10 years disease-free and overall survival rates when compared with pdHCC patients (Figure 1). When we analyze the recurrence rates, there was only 1 patient who had tumor recurrence in the iHCC group, while 76 patients had tumor recurrence in the pdHCC group (1.85% vs. 21%, *p* = 0.001) (Figure 1).

**Figure 2:**
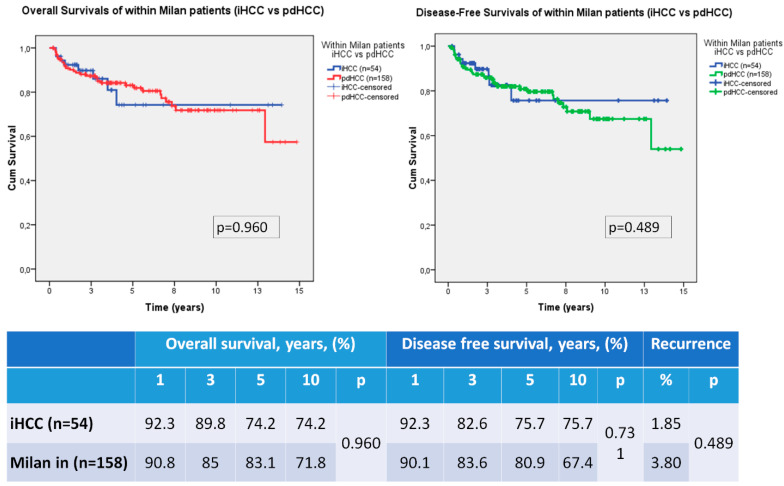
Survival comparison of Hepatocellular carcinoma patients (iHCC vs. within Milan in pdHCC).

Then, we compared the survival and recurrence rates of the iHCC patients (*n* = 54) versus within the Milan criteria in the pdHCC patients (*n* = 158). There was no DFS or OS difference between the iHCC patients and dHCC patients who met the Milan criteria. There was no statistically significant difference in tumor recurrence rates between these two groups (Figure 2).

**Figure 3:**
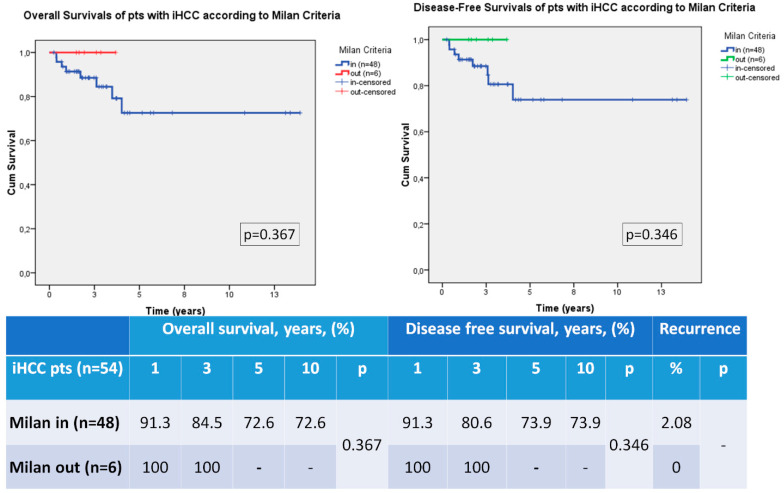
Survival comparison of iHCC patients (iHCC in Milan vs. iHCC out of Milan).

Lastly, we analyzed the survival and recurrence rates of the iHCC patients according to the Milan criteria. There was no significant difference in terms of DFS and OS rates. There were only six patients who did not meet the Milan criteria in iHCC group, so statistical evaluation could not reach significant value (Figure 3).

## 4. Discussion

The incidence of iHCC in our series was 1.6% (54/3204), which is lower than previously reported series (4.2% to 40%) [5,6,7,8,9]. If we observed suspicious nodules in the liver during the pre-transplant evaluation of the transplant candidate we routinely performed liver-specific contrast-enhanced MRI in order to identify tumor presence. We think that this approach contributed to the low incidence rate of iHCC in our series. Only 54 out of 406 HCC patients were diagnosed after the histopathologic examination of the explanted livers. Demographic features, such as age, gender and body mass index, do not have any significant difference between the pdHCC and iHCC patients in our series.

The etiology of the underlying liver disease between pdHCC and iHCC groups had some differences in our study population. Viral hepatitis were statistically more frequent in the pdHCC group (83%) than in the iHCC group (59.3%). We think that the interaction between viral replication and tumor growth may be related to this result. Nevertheless, cryptogenic cirrhosis and Budd–Chiari syndrome are more frequent underlying liver diseases in the iHCC group. We think that the appearance of macronodular cirrhosis in the imaging of Budd–Chiari patients may increase the rate of iHCC by superposing small tumors. In the previous reports, HCV hepatitis is higher in iHCC patients [10,11]. Although we cannot explain the reason with strong evidence-based data, it is the first study to show that iHCC patients may differ from pdHCC patients in terms of etiological features.

MELD/PELD scores of iHCC patients were higher than pdHCC patients in our study. When we compare the Child–Pugh scores of these two groups, Child–Pugh A patients were much higher in the pdHCC group (37.8% vs. 5.6%), and even the Child–Pugh C patients were higher in the iHCC group (44.4% vs. 19.9%). In our study, pdHCC patients had better liver function than iHCC patients. Worsened liver function, inflammation, severe liver fibrosis and regeneration are particular features in the pathogenesis of HCC, and these features were also reported in the previous studies about iHCC [12,13,14,15,16]. So, patients with advanced cirrhosis may be included into a specific surveillance protocol that consist of more frequent high resolution imaging and biopsy of suspicious nodules in order to diagnose missed HCC’s.

AFP levels calculated before liver transplantation were higher in pdHCC patients, as expected, because of the multifocal and larger tumors. Additionally, GGT levels were higher in the pdHCC group as a proinflammatory marker.

In our study population, most of the iHCC patients had uninodular lesions (66.7%); on the other hand, pdHCC group had more multinodular tumors (52.8%). Additionally, maximum tumor diameter was statistically higher in pdHCC group. In the pdHCC group, patients had more poor and moderate tumor differentiation, but in the iHCC group patients had more well and moderately differentiated tumors. There was no macrovascular invasion and only 7% microvascular invasion in İHCC patients. In contrast to our study, Perez et al. reported that the predominant part of the iHCC patients had multinodular tumors (55.6%) and they had macrovascular (3.7%) and microvascular invasion (14.8%). They also had more poorly and moderately differentiated iHCC tumors (70.4%) [11]. On the contrary, Rajakannu et al. reported that majority of iHCC’s have well differentiated tumors without micro/macrovascular invasion within the Milan criteria, similar to our study [17]. We should also mention that in our study, most of the iHCC tumors were within the Milan criteria as well (88.9%).

In our study, iHCC recurrence rate was only 1.85 %; this may be due to our iHCC patients’ tumor characteristics, as we found that iHCC patients had mostly uninodular, relatively small tumors within the Milan criteria and also microvascular invasion rate was only 7.4% versus 38% in the larger diameter pdHCC group. Some previous reports about iHCC found similar tumor features with negligible tumor recurrence [6,8]. Perez et al. reported that iHCC tumors often had poor histopathological features beyond the Milan criteria. They mentioned that inadequate selection and prioritization of candidates without bridging locoregional therapies for iHCC patients have similar recurrence rates with pdHCC [11].

OS and DFS rates at 1, 3, 5 and 10 years were better in iHCC patients than pdHCC patients in our series (Figure 1). It may be due to better histological features and low recurrence rate in our series. Most of the iHCC patients of our study group were within the Milan criteria, so we compared them with pdHCC patients who met the Milan criteria. DFS, OS and also recurrence rates were not statistically different (Figure 2). Thus, we believe that iHCC tumor characteristics may be similar with pdHCC patients who met the Milan criteria. Finally, we also compared the survival rates of iHCC patients who met the Milan criteria and iHCC patients out of the Milan criteria. There were only six patients who did not meet the Milan criteria in the iHCC group and interestingly, they were still alive and had no recurrence. Statistical evaluation could not reach any significant result because of the small sample size (Figure 3). In addition, the Milan criteria patients in the iHCC group with multiple tumors had a poor prognostic factor, but these patients had at least 3 years OS and DFS 100% of the time. Additionally, some previous reports about liver transplantation criteria for HCC ignore the number of tumors [17,18,19,20,21].

The limitations of this study include its retrospective design and the relatively small number of patients. Meanwhile, the strengths of this study include the use of the largest volume of the European liver transplant center’s databank, which has each transplant patient consecutively recorded in a prospective manner, and being the first study that compares the survivals of iHCC versus pdHCC within the Milan criteria.

## 5. Conclusions

The iHCC tumors showed better histopathologic features than pdHCC with a low recurrence rate. The iHCC patients had better survival than pdHCC patients. Moreover, iHCC patients had similar survival and recurrence rates compared with patients in the pdHCC group who met the Milan criteria. The other unique finding of our study is the significant difference in underlying liver disease between the iHCC and pdHCC patients. Further investigations are needed on this subject, with larger numbers of patient.

## Figures and Tables

**Table 1:** Clinical characteristics and demographic features of pdHCC and iHCC.

Variables (*n*, %)	pdHCC (*n* = 352)	iHCC (*n* = 54)	*p*-Value
Age [Median (min.-max.)]	56 (4-72)	53.5 (2-72)	0.047
Gender Male Female	303 (86.1) 49 (13.9)	46 (85.2) 8 (14.8)	1.000
BMI [Median (min.-max.)]	25.7 (16.3-46.9)	26.9 (16.4-41)	0.474
Underlying liver disease Viral hepatitis Cryptogenic cirrhosis Budd–Chiari syndrome Alcoholic liver disease Metabolic liver diease NASH Others	292 (83) ^a^ 40 (11.4) ^a^ 5 (1.4) ^a^ 4 (1.1) 4 (1.1) 2 (0.6) 5 (1.4)	32 (59.3) ^b^ 12 (22.2) ^b^ 4 (7.4) ^b^ 2 (3.7) 2 (3.7) 1 (1.9) 1(1.9)	0.012
MELD/ PELD score [Median (min.–max.)]	12 (6-41)	16.5 (9-31)	<0.001
Child–Pugh Score A B C	133 (37.8) ^a^ 149 (42.3) 70 (19.9) ^a^	3 (5.6) ^b^ 27 (50.0) 24 (44.4) ^b^	<0.001
PreTx **AFP** (ng/mL), [Median (min.–max.)]	15.3 (0.2-20,179)	4.45 (0.4-1001)	<0.001
**GGT** (IU/mL), [Median (min.–max.)]	74 (11-1396)	49.5 (13-349)	0.001

^a^ and ^b^ superscripts indicate the statistically significant difference between groups. Abbreviations; iHCC: incidentally diagnosed hepatocellular carcinoma, pdHCC: preoperatively diagnosed hepatocellular carcinoma, MELD: model for end stage liver disease score, PELD: pediatric end stage liver disease score.

**Table 2:** Histopathological features of iHCC and pdHCC.

Variables (*n*, %)	pdHCC (*n* = 352)	iHCC (*n* = 54)	*p*-Value
Number of nodules Uninodular Multinodular	166 (47.2) 186 (52.8)	36 (66.7) 18 (33.3)	0.012
MTD (cm) [Median (min.–max.)]	3.5 (0.1-24)	1 (0.1-2.5)	<0.001
Tm differentiation Well Moderate Poor	127 (36.1) ^a^ 161 (45.7) ^a^ 64 (18.2) ^a^	43 (79.6) ^b^ 10 (18.5) ^b^ 1 (1.9) ^b^	<0.001
Vascular invasion Macrovascular inv. Microvascular inv. No vascular invasion	48 (13.6) ^a^ 135 (38.4) ^a^ 169 (48.0) ^a^	0 (0) ^b^ 4 (7.4) ^b^ 50 (92.6) ^b^	<0.001
Milan criteria Within Beyond	158 (44.9) 194 (55.1)	48 (88.9) 6 (11.1)	<0.001
Locoregional therapy pre-LT	71 (20.2)	0 (0)	<0.001

^a^ and ^b^ superscripts indicate the statistically significant difference between groups. Abbreviations; iHCC: incidentally diagnosed hepatocellular carcinoma, pdHCC: preoperatively diagnosed hepatocellular carcinoma, MTD: maximum tumor diameter.

## Data Availability

All the data can be achieved from our institute.

## Abbreviations

**HCC**: hepatocellular carcinoma, **iHCC**: incidental hepatocellular carcinoma, **pdHCC**: preoperatively diagnosed hepatocellular carcinoma, **DFS**: disease free survival, **OS**: overall survival, **AFP**: alpha feto protein, **GGT**: gamma glutamyl transferase, **CT**: computed tomography, **MRI**: magnetic resonance imaging, **BMI**: body mass index, **NASH**: non-alcoholic steatohepatitis, **LT**: Liver transplantation, **MTD**: maximum tumor diameter, **MELD**: model for end stage liver disease score, **PELD**: pediatric end stage liver disease score.
